# Supplementary material for: Role of N-Cadherin in Epithelial-to-Mesenchymal Transition and Chemosensitivity of Colon Carcinoma Cells
Source: Cancers (Basel). 2022 Oct 20;14(20):5146. doi: 10.3390/cancers14205146 (PMC9601123; doi:10.3390/cancers14205146)
Supplement: Supplementary file 1 [file cancers-14-05146-s001.zip › Supplementary material_miRNOM.pdf]

Supplementary Material

Supplementary Table S2: Differentially expressed miRNA (|FC| ≥ 2) between HCT8 NC and HCT8 CTRL tumor samples

miRNA name	adj. *p* value	*p* value	Fold change (HCT8 NC/HCT8 CTRL)	Regulation	miRBase accession number
**hsa-miR-15b-5p**	NS	NS	2.37	up	MIMAT0000417
**hsa-let-7g-5p**	NS	NS	2.33	up	MIMAT0000414
**hsa-let-7f-5p**	NS	NS	2.33	up	MIMAT0000067
**hsa-let-7b-5p**	NS	NS	2.31	up	MIMAT0000063
**hsa-miR-199a-5p**	NS	NS	2.26	up	MIMAT0000231
**hsa-let-7c-5p**	NS	NS	2.24	up	MIMAT0000064
**hsa-let-7a-5p**	NS	NS	2.20	up	MIMAT0000062
**hsa-let-7d-5p**	NS	NS	2.17	up	MIMAT0000065
**hsa-miR-27a-3p**	NS	NS	2.14	up	MIMAT0000084
**hsa-miR-21-5p**	NS	NS	2.11	up	MIMAT0000076
**hsa-miR-215-5p**	NS	NS	2.08	up	MIMAT0000272
**hsa-miR-20a-5p**	NS	NS	2.07	up	MIMAT0000075
**hsa-let-7i-5p**	NS	NS	2.07	up	MIMAT0000415
**hsa-miR-194-5p**	NS	NS	2.03	up	MIMAT0000460
**hsa-miR-29c-3p**	NS	NS	2.03	up	MIMAT0000681
**hsa-miR-203a-3p**	NS	NS	2.02	up	MIMAT0000264
**hsa-miR-5001-5p**	NS	NS	-2.02	down	MIMAT0021021
**hsa-miR-765**	NS	NS	-2.10	down	MIMAT0003945
**hsa-miR-4446-3p**	NS	NS	-2.15	down	MIMAT0018965
**hsa-miR-619-5p**	NS	NS	-2.19	down	MIMAT0026622
**hsa-miR-6858-5p**	NS	NS	-2.35	down	MIMAT0027616
**hsa-miR-3620-5p**	2.92E-10	7.17E-13	-28.52	down*	MIMAT0022967

NS = not significant
* miRNAs not detected in HCT8 NC tumor samples

Supplementary Table S3: Differentially expressed miRNA (|FC| ≥ 2) between HCT8 NC and HCT8 CTRL plasma samples

miRNA name	adj. *p* value	*p* value	Fold change (HCT8 NC/HCT8 CTRL)	Regulation	miRBase accession number
**hsa-miR-6884-3p**	NS	NS	2.17	up	MIMAT0027669
**hsa-miR-494-3p**	NS	NS	-2.01	down	MIMAT0002816
**hsa-miR-122-5p**	NS	NS	-2.01	down	MIMAT0000421
**hsa-miR-3680-3p**	8.91E-07	1.55E-09	-20.57	down*	MIMAT0018107

NS = not significant
* miRNAs not detected in HCT8 NC tumor samples

Supplementary Table S4: Experimentally validated targets (N = 57) of hsa-miR-3620-p5 according to TarBase v8 and miRTarBase v8 databases

Gene symbol	Entrez	Validation method	Pubmed ID
**ABCG8**	64241	PAR-CLIP	27292025
**ADGRL1**	22859	PAR-CLIP	26701625
**AKT1S1**	84335	PAR-CLIP	26701625
**B4GALT7**	11285	HITS-CLIP	23313552
**BARX2**	8538	PAR-CLIP	26701625
**CCNF**	899	PAR-CLIP	23592263
**CLDN4**	1364	PAR-CLIP	26701625
**COL1A1**	1277	PAR-CLIP	26701625
**COX6B1**	1340	HITS-CLIP	23313552
**DAO**	1610	HITS-CLIP	19536157
**DMRT2**	10655	PAR-CLIP	20371350
**DRAXIN**	374946	HITS-CLIP	23824327
**ELK1**	2002	HITS-CLIP	23824327
**FADS6**	283985	PAR-CLIP	22012620
**FBXW8**	26259	PAR-CLIP	26701625
**FGF19**	9965	PAR-CLIP	26701625
**GDE1**	51573	HITS-CLIP	23824327
**GPX1**	2876	PAR-CLIP	23446348|20371350
**HIST1H1E**	3008	PAR-CLIP	23446348
**HLA-B**	3106	HITS-CLIP	19536157
**CHST3**	9469	HITS-CLIP	23824327
**INAFM1**	255783	PAR-CLIP	26701625
**IPCEF1**	26034	HITS-CLIP	23824327
**KBTBD12**	166348	PAR-CLIP	23592263|27292025
**LAX1**	54900	HITS-CLIP	23824327
**MAP1S**	55201	PAR-CLIP	26701625
**MED28**	80306	HITS-CLIP	23824327
**NPTX1**	4884	HITS-CLIP	23824327
**ORC6**	23594	PAR-CLIP	27292025
**PHF19**	26147	PAR-CLIP	23592263
**PKM**	5315	PAR-CLIP	26701625
**PLXND1**	23129	PAR-CLIP	23592263
**PNRC2**	55629	PAR-CLIP	21572407
**POM121C**	100101267	PAR-CLIP	23592263
**POU2F3**	25833	HITS-CLIP	23824327|23313552
**PPM1N**	147699	HITS-CLIP	23824327
**RAD54L2**	23132	HITS-CLIP	23824327|23313552
**RNF4**	6047	PAR-CLIP	20371350|26701625
**SKI**	6497	PAR-CLIP	27292025
**SLC12A7**	10723	PAR-CLIP	27292025
**SLC2A8**	29988	HITS-CLIP	19536157
**SLC35C2**	51006	PAR-CLIP	23592263
**SLC39A11**	201266	HITS-CLIP	19536157
**SLC6A8**	6535	HITS-CLIP	23824327

**SLC7A1**	6541	PAR-CLIP	26701625
**SPA17**	53340	HITS-CLIP	19536157
**SPTBN2**	6712	PAR-CLIP	23446348|21572407
**TAF8**	129685	PAR-CLIP	20371350
**TIAL1**	7073	HITS-CLIP	23824327
**TIMM8B**	26521	PAR-CLIP	22100165
**TMEM91**	641649	PAR-CLIP	23592263
**TRIM28**	10155	PAR-CLIP	26701625
**TUBB2A**	7280	PAR-CLIP	20371350
**UBALD2**	283991	PAR-CLIP	27292025
**WARS**	7453	HITS-CLIP	23313552
**ZMAT1**	84460	HITS-CLIP	23824327
**ZSCAN25**	221785	HITS-CLIP	19536157

Supplementary Table S5: Experimentally validated targets (N = 178) of hsa-miR-3680-3p according to TarBase v8 and miRTarBase v8 databases

Gene symbol	Entrez ID	Validation method	Pubmed ID
**AAGAB**	79719	HITS-CLIP	23824327
**ABCC12**	94160	HITS-CLIP	23824327
**ACER3**	55331	HITS-CLIP	27418678
**ACSL4**	2182	HITS-CLIP//PAR-CLIP	21572407
**ACSS3**	79611	PAR-CLIP	22012620
**ACTB**	60	PAR-CLIP	20371350
**ACVR1B**	91	PAR-CLIP	23446348|26701625
**ADAM19**	8728	HITS-CLIP	23824327
**AGO2**	27161	PAR-CLIP	22012620|21572407
**AKAP10**	11216	PAR-CLIP	20371350
**AKR7A2**	8574	PAR-CLIP	20371350
**ALDH6A1**	4329	HITS-CLIP	23313552
**ALKBH5**	54890	PAR-CLIP	20371350
**ANKEF1**	63926	HITS-CLIP//PAR-CLIP	23592263|24398324|23446348|22012620|21572407|20371350|23706177|23313552|27292025
**ARHGAP1**	392	HITS-CLIP	23824327
**ARHGAP31**	57514	HITS-CLIP	23824327
**ARHGEF3**	50650	PAR-CLIP	22291592
**ARID1A**	8289	PAR-CLIP	20371350
**AUTS8**	282553	HITS-CLIP	23824327
**AZF1**	560	PAR-CLIP	22012620|20371350
**BACH1**	571	HITS-CLIP//PAR-CLIP	20371350|23824327
**BCL7A**	605	PAR-CLIP	20371350
**BID**	637	HITS-CLIP	23824327
**BOD1**	91272	HITS-CLIP	23824327|27418678|28735896
**BRD4**	23476	HITS-CLIP	23824327
**BUB1**	699	PAR-CLIP	22012620
**C4orf26**	152816	HITS-CLIP	19536157
**CALM1**	801	PAR-CLIP	23446348|21572407|20371350

**CALM2**	805	HITS-CLIP//PAR-CLIP	23592263|23446348|20371350|21572407
**CASP3**	836	PAR-CLIP	23592263
**CBX4**	8535	HITS-CLIP	23824327|27418678
**CCDC39**	339829	HITS-CLIP	23824327
**CCNT2**	905	PAR-CLIP	24398324
**CDC42EP3**	10602	HITS-CLIP	27418678
**CDH6**	1004	HITS-CLIP	23824327|27418678
**CENPK**	64105	HITS-CLIP	23313552
**CERK**	64781	HITS-CLIP	23313552
**CFAP20**	29105	HITS-CLIP	28735896
**CLOCK**	9575	HITS-CLIP	23824327
**CNBP**	7555	PAR-CLIP	22012620
**COL9A2**	1298	HITS-CLIP	23824327
**COLEC10**	10584	PAR-CLIP	21572407
**CRIPT**	9419	HITS-CLIP	23824327
**CRK**	1398	PAR-CLIP	27292025
**CTNS**	1497	HITS-CLIP	23824327
**DNAJC15**	29103	HITS-CLIP	23313552|27418678
**DR1**	1810	HITS-CLIP	28735896
**DUSP14**	11072	PAR-CLIP	21572407
**DUSP8**	1850	PAR-CLIP	21572407
**DYM**	54808	HITS-CLIP	23824327
**EDN1**	1906	HITS-CLIP	23824327
**EI24**	9538	HITS-CLIP	23824327|27418678
**EOGT**	285203	HITS-CLIP	27418678
**ETS1**	2113	HITS-CLIP	23313552
**FAM210B**	116151	HITS-CLIP	27418678
**FAM46C**	54855	PAR-CLIP	21572407
**FAM9C**	171484	HITS-CLIP	23824327
**FER**	2241	HITS-CLIP	23824327
**FNIP2**	57600	HITS-CLIP	23824327
**FOXP2**	93986	HITS-CLIP	27418678
**FRK**	2444	PAR-CLIP	24398324
**FST**	10468	HITS-CLIP	27418678
**GABBR2**	9568	HITS-CLIP	23824327|27418678
**GDNF**	2668	PAR-CLIP	21572407|20371350
**GLCCI1**	113263	HITS-CLIP	27418678
**GM2A**	2760	PAR-CLIP	22100165
**GOLPH3**	64083	HITS-CLIP	19536157
**GPATCH8**	23131	HITS-CLIP	23824327
**GPC4**	2239	PAR-CLIP	21572407|20371350
**GTF2H1**	2965	HITS-CLIP	23824327
**GUCD1**	83606	PAR-CLIP	23446348|20371350
**HBS1L**	10767	HITS-CLIP	23824327|27418678
**HEG1**	57493	HITS-CLIP	27418678
**HMGB1**	3146	HITS-CLIP	23824327
**HMGXB4**	10042	PAR-CLIP	21572407
**HNRNPA3**	220988	HITS-CLIP	19536157
**HNRNPF**	3185	PAR-CLIP	24398324|26701625|27292025

**HSD17B12**	51144	HITS-CLIP	23824327
**CHIC1**	53344	PAR-CLIP	21572407
**IMP4**	92856	HITS-CLIP	19536157
**JCAD**	57608	HITS-CLIP	23824327
**KANK4**	163782	HITS-CLIP	23824327
**KCNIP3**	30818	HITS-CLIP	23824327
**KCNJ6**	3763	HITS-CLIP	23824327
**KCTD16**	57528	HITS-CLIP	27418678
**KIAA0408**	9729	PAR-CLIP	21572407
**KLF7**	8609	HITS-CLIP	23824327
**LIMS1**	3987	PAR-CLIP	20371350
**LRRC40**	55631	HITS-CLIP	23824327
**LRRC55**	219527	HITS-CLIP//PAR-CLIP	23446348|21572407|20371350|23706177|27292025
**MACC1**	346389	HITS-CLIP	23824327|23313552
**MAGEA3**	4102	PAR-CLIP	27292025
**MAGEA6**	4105	PAR-CLIP	27292025
**MAPK8**	5599	PAR-CLIP	24398324|22012620
**MED24**	9862	HITS-CLIP	23824327|22927820
**MLLT6**	4302	HITS-CLIP	23824327
**MMAB**	326625	HITS-CLIP	23824327
**MRPS10**	55173	HITS-CLIP	21572407
**MTRNR2L6**	100463482	PAR-CLIP	23446348
**MYO10**	4651	PAR-CLIP	22012620|21572407
**NRARP**	441478	PAR-CLIP	20371350
**NWD1**	284434	PAR-CLIP	21572407
**ONECUT3**	390874	HITS-CLIP	23824327
**P2RY1**	5028	HITS-CLIP	23824327|23313552
**PARP15**	165631	HITS-CLIP//PAR-CLIP	24398324|23313552
**PDE3A**	5139	PAR-CLIP	22100165
**PEX26**	55670	HITS-CLIP	23824327
**PHIP**	55023	HITS-CLIP	27418678
**PITPNC1**	26207	PAR-CLIP	21572407
**PLIN1**	5346	HITS-CLIP	23824327
**POGK**	57645	PAR-CLIP	20371350
**POTED**	317754	HITS-CLIP	23824327|27418678|28735896
**PRRC2B**	84726	HITS-CLIP	23824327
**PTCD2**	79810	HITS-CLIP	23824327
**PTPRM**	5797	HITS-CLIP	23824327
**PURB**	5814	PAR-CLIP	23592263
**RAB10**	10890	PAR-CLIP	21572407|20371350|26701625
**RAET1E**	135250	HITS-CLIP	23824327
**RBBP9**	10741	PAR-CLIP	23446348
**RBM47**	54502	HITS-CLIP	23824327
**RBPJ**	3516	PAR-CLIP	26701625
**RDH13**	112724	PAR-CLIP	22012620
**REEP1**	65055	HITS-CLIP	23824327|27418678
**REL**	5966	HITS-CLIP	23824327
**RLIM**	51132	PAR-CLIP	21572407

**RPL30**	6156	PAR-CLIP	23592263
**RPLP0**	6175	PAR-CLIP	20371350
**RPS6KA5**	9252	HITS-CLIP	27418678
**RRP7A**	27341	PAR-CLIP	20371350
**RYBP**	23429	HITS-CLIP	23824327
**SATB1**	6304	PAR-CLIP	23446348|21572407
**SBNO1**	55206	HITS-CLIP	23824327
**SCML2**	10389	HITS-CLIP	23824327
**SEMA6A**	57556	PAR-CLIP	23592263
**SEPT8**	23176	PAR-CLIP	23592263
**SESN3**	143686	PAR-CLIP	20371350
**SF3A1**	10291	HITS-CLIP	23824327
**SKI**	6497	PAR-CLIP	20371350
**SKIL**	6498	PAR-CLIP	22012620
**SLBP**	7884	PAR-CLIP	21572407
**SLC16A1**	6566	PAR-CLIP	23592263|21572407
**SLC30A10**	55532	HITS-CLIP	23824327
**SLC43A2**	124935	HITS-CLIP	23824327
**SLC7A2**	6542	HITS-CLIP	23824327
**SMU1**	55234	HITS-CLIP//PAR-CLIP	22012620|21572407|23706177|26701625|27292025
**SNED1**	25992	HITS-CLIP	19536157
**SNTB2**	6645	PAR-CLIP	22012620|21572407
**SPIN4**	139886	HITS-CLIP	23313552
**SPRY1**	10252	HITS-CLIP//PAR-CLIP	21572407
**ST8SIA3**	51046	HITS-CLIP	23824327
**STARD3NL**	83930	HITS-CLIP	23824327
**STRN3**	29966	HITS-CLIP//PAR-CLIP	21572407|20371350|23706177
**TBXA2R**	6915	HITS-CLIP	23824327
**TIAM2**	26230	HITS-CLIP	27418678
**TMCC1**	23023	PAR-CLIP	20371350
**TMEM245**	23731	HITS-CLIP	27418678
**TMEM251**	26175	HITS-CLIP	27418678
**TMEM59**	9528	HITS-CLIP	23824327
**TMEM64**	169200	PAR-CLIP	23592263
**UBTF**	7343	HITS-CLIP	23824327
**UNC5D**	137970	HITS-CLIP	23824327
**USP6NL**	9712	PAR-CLIP	23446348
**VCAN**	1462	HITS-CLIP	27418678
**WAC**	51322	HITS-CLIP	27418678
**WISP1**	8840	HITS-CLIP	23824327
**XIAP**	331	HITS-CLIP	23824327
**YIPF6**	286451	PAR-CLIP	22012620
**ZBTB5**	9925	PAR-CLIP	22012620
**ZNF154**	7710	HITS-CLIP//PAR-CLIP	23446348|21572407|20371350
**ZNF208**	7757	HITS-CLIP	23313552
**ZNF226**	7769	PAR-CLIP	23446348|20371350
**ZNF25**	219749	HITS-CLIP	23824327
**ZNF410**	57862	PAR-CLIP	21572407

**ZNF525**	170958	PAR-CLIP	22100165
**ZNF701**	55762	PAR-CLIP	22100165
**ZNF703**	80139	PAR-CLIP	23446348|20371350
**ZNF813**	126017	PAR-CLIP	20371350
**ZNRF2**	223082	HITS-CLIP//PAR-CLIP	23446348|21572407|20371350|27292025

Supplementary Table S6: Differentially expressed miRNA (|FC| ≥ 2, adj. *p* < 0.05) between colon cancer primocultures T47B and T48B

miRNA name	adj. *p* value	*p* value	Regulation	Fold change T47B/T48B	miRBase accession number
**hsa-miR-3135b**	1.65E-04	2.61E-05	up	269.23*	MIMAT0018985
**hsa-miR-3654**	3.08E-04	5.20E-05	up	160.63*	MIMAT0018074
**hsa-miR-4499**	4.07E-06	2.10E-08	up	151.57*	MIMAT0019035
**hsa-miR-6887-5p**	2.74E-04	4.43E-05	up	145.95*	MIMAT0027674
**hsa-miR-218-5p**	3.06E-05	3.11E-06	up	134.56*	MIMAT0000275
**hsa-miR-4800-5p**	1.21E-05	7.67E-07	up	114.62*	MIMAT0019978
**hsa-miR-134-5p**	7.28E-05	9.57E-06	up	114.55*	MIMAT0000447
**hsa-miR-4478**	4.64E-05	5.06E-06	up	113.68*	MIMAT0019006
**hsa-miR-1181**	1.21E-05	7.97E-07	up	109.64*	MIMAT0005826
**hsa-miR-6833-5p**	1.53E-04	2.29E-05	up	105.35*	MIMAT0027566
**hsa-miR-3141**	6.64E-05	8.37E-06	up	100.14*	MIMAT0015010
**hsa-miR-7847-3p**	2.98E-05	2.81E-06	up	96.58*	MIMAT0030422
**hsa-miR-3663-3p**	2.98E-05	2.40E-06	up	90.73*	MIMAT0018085
**hsa-miR-142-5p**	6.64E-05	8.08E-06	up	88.70*	MIMAT0000433
**hsa-miR-135a-3p**	1.09E-03	2.86E-04	up	87.60*	MIMAT0004595
**hsa-miR-4291**	1.14E-05	5.99E-07	up	84.91*	MIMAT0016922
**hsa-miR-6867-5p**	5.35E-05	6.03E-06	up	82.32*	MIMAT0027634
**hsa-miR-6850-5p**	4.31E-06	7.99E-08	up	80.10*	MIMAT0027600
**hsa-miR-6812-5p**	2.98E-05	2.84E-06	up	78.45*	MIMAT0027524
**hsa-miR-6752-5p**	2.98E-05	2.53E-06	up	71.23*	MIMAT0027404
**hsa-miR-7108-5p**	4.07E-06	4.43E-08	up	65.75*	MIMAT0028113
**hsa-miR-4298**	4.07E-06	4.59E-08	up	65.22*	MIMAT0016852
**hsa-miR-6791-5p**	2.45E-03	8.00E-04	up	36.52*	MIMAT0027482
**hsa-miR-3652**	4.56E-06	1.85E-07	up	23.16*	MIMAT0018072
**hsa-miR-8060**	4.56E-06	1.89E-07	up	22.80*	MIMAT0030987
**hsa-miR-6756-5p**	4.85E-06	2.19E-07	up	22.32*	MIMAT0027412
**hsa-miR-4778-5p**	1.21E-05	7.36E-07	up	22.22*	MIMAT0019936
**hsa-miR-6840-3p**	3.08E-04	5.29E-05	up	21.32*	MIMAT0027583
**hsa-miR-1249-3p**	8.27E-05	1.15E-05	up	21.13*	MIMAT0005901
**hsa-miR-7107-5p**	3.08E-03	1.11E-03	up	4.95	MIMAT0028111
**hsa-miR-6723-5p**	7.23E-03	3.39E-03	up	4.13	MIMAT0025855
**hsa-miR-4485-3p**	9.37E-04	2.25E-04	up	4.04	MIMAT0019019

**hsa-miR-6724-5p**	4.89E-03	2.02E-03	up	3.59	MIMAT0025856
**hsa-miR-424-5p**	4.41E-02	3.12E-02	up	3.39	MIMAT0001341
**hsa-miR-5787**	1.81E-02	1.09E-02	up	3.27	MIMAT0023252
**hsa-miR-4281**	1.03E-03	2.63E-04	up	3.19	MIMAT0016907
**hsa-miR-4459**	6.93E-03	3.18E-03	up	3.17	MIMAT0018981
**hsa-miR-4516**	1.68E-03	4.80E-04	up	2.95	MIMAT0019053
**hsa-miR-188-5p**	9.25E-04	2.18E-04	up	2.82	MIMAT0000457
**hsa-miR-1973**	4.86E-04	9.50E-05	up	2.76	MIMAT0009448
**hsa-miR-8069**	1.60E-04	2.47E-05	up	2.62	MIMAT0030996
**hsa-miR-7110-5p**	3.04E-03	1.06E-03	up	2.57	MIMAT0028117
**hsa-miR-6869-5p**	3.61E-03	1.44E-03	up	2.53	MIMAT0027638
**hsa-miR-1225-5p**	9.69E-04	2.40E-04	up	2.53	MIMAT0005572
**hsa-miR-7150**	9.75E-03	5.13E-03	up	2.52	MIMAT0028211
**hsa-miR-125b-5p**	2.96E-03	1.02E-03	up	2.52	MIMAT0000423
**hsa-miR-6769b-5p**	1.39E-02	8.07E-03	up	2.48	MIMAT0027620
**hsa-miR-22-3p**	2.22E-03	7.00E-04	up	2.42	MIMAT0000077
**hsa-miR-642a-3p**	2.22E-03	7.02E-04	up	2.42	MIMAT0020924
**hsa-miR-6891-5p**	3.72E-02	2.56E-02	up	2.33	MIMAT0027682
**hsa-miR-8063**	3.82E-02	2.65E-02	up	2.29	MIMAT0030990
**hsa-miR-1207-5p**	3.54E-03	1.39E-03	up	2.28	MIMAT0005871
**hsa-miR-6821-5p**	8.74E-03	4.37E-03	up	2.27	MIMAT0027542
**hsa-miR-24-3p**	3.54E-03	1.38E-03	up	2.24	MIMAT0000080
**hsa-miR-1587**	3.56E-03	1.40E-03	up	2.13	MIMAT0019077
**hsa-miR-642b-3p**	5.68E-03	2.44E-03	up	2.11	MIMAT0018444
**hsa-miR-1275**	1.32E-04	1.93E-05	up	2.11	MIMAT0005929
**hsa-miR-6090**	2.06E-02	1.25E-02	up	2.10	MIMAT0023715
**hsa-miR-142-3p**	9.08E-03	4.61E-03	up	2.10	MIMAT0000434
**hsa-miR-4687-3p**	5.91E-03	2.58E-03	up	2.08	MIMAT0019775
**hsa-miR-6088**	5.22E-03	2.18E-03	up	2.02	MIMAT0023713
**hsa-miR-197-5p**	1.39E-02	8.00E-03	down	-2.02	MIMAT0022691
**hsa-miR-6131**	9.25E-04	2.19E-04	down	-2.04	MIMAT0024615
**hsa-miR-200a-3p**	5.76E-04	1.17E-04	down	-2.09	MIMAT0000682
**hsa-miR-125a-5p**	2.22E-03	6.94E-04	down	-2.10	MIMAT0000443
**hsa-miR-148a-3p**	6.23E-03	2.74E-03	down	-2.11	MIMAT0000243
**hsa-miR-374a-5p**	1.41E-02	8.29E-03	down	-2.13	MIMAT0000727
**hsa-miR-5581-5p**	1.14E-03	3.09E-04	down	-2.17	MIMAT0022275
**hsa-miR-4716-3p**	2.52E-03	8.34E-04	down	-2.18	MIMAT0019827
**hsa-miR-4728-5p**	9.63E-04	2.35E-04	down	-2.24	MIMAT0019849
**hsa-miR-1273g-3p**	5.42E-03	2.28E-03	down	-2.28	MIMAT0022742
**hsa-miR-331-3p**	1.03E-03	2.61E-04	down	-2.29	MIMAT0000760
**hsa-miR-5088-5p**	5.68E-03	2.46E-03	down	-2.34	MIMAT0021080
**hsa-miR-3125**	3.54E-03	1.33E-03	down	-2.39	MIMAT0014988
**hsa-miR-30c-5p**	8.64E-04	1.98E-04	down	-2.42	MIMAT0000244

**hsa-miR-130b-3p**	2.12E-03	6.38E-04	down	-2.68	MIMAT0000691
**hsa-miR-19a-3p**	1.75E-03	5.08E-04	down	-2.68	MIMAT0000073
**hsa-let-7a-5p**	2.40E-02	1.52E-02	down	-2.76	MIMAT0000062
**hsa-miR-324-5p**	5.30E-04	1.06E-04	down	-3.23	MIMAT0000761
**hsa-miR-4746-3p**	6.35E-04	1.36E-04	down	-3.29	MIMAT0019881
**hsa-let-7e-5p**	1.82E-03	5.41E-04	down	-3.41	MIMAT0000066
**hsa-miR-20a-5p**	3.46E-04	6.38E-05	down	-3.70	MIMAT0000075
**hsa-miR-203a-3p**	2.41E-03	7.71E-04	down	-4.16	MIMAT0000264
**hsa-miR-17-5p**	2.36E-05	1.77E-06	down	-4.33	MIMAT0000070
**hsa-miR-1914-3p**	3.19E-04	5.64E-05	down	-4.73	MIMAT0007890
**hsa-miR-20b-5p**	3.04E-03	1.07E-03	down	-4.85	MIMAT0001413
**hsa-miR-4497**	3.52E-04	6.68E-05	down	-7.59	MIMAT0019032
**hsa-miR-4299**	3.08E-04	5.32E-05	down	-7.72	MIMAT0016851
**hsa-miR-301a-3p**	7.16E-04	1.59E-04	down	-14.23	MIMAT0000688
**hsa-miR-338-5p**	8.59E-03	4.26E-03	down	-24.07	MIMAT0004701
**hsa-miR-221-3p**	1.07E-04	1.53E-05	down	-24.79	MIMAT0000278
**hsa-miR-146b-5p**	5.88E-05	6.85E-06	down	-27.89	MIMAT0002809
**hsa-miR-30e-3p**	3.54E-03	1.36E-03	down	-41.88*	MIMAT0000693
**hsa-miR-151a-5p**	2.98E-05	2.92E-06	down	-45.51*	MIMAT0004697
**hsa-miR-148b-3p**	2.69E-03	9.09E-04	down	-56.57*	MIMAT0000759
**hsa-miR-590-5p**	2.59E-03	8.66E-04	down	-58.94*	MIMAT0003258
**hsa-miR-532-3p**	3.52E-04	6.75E-05	down	-73.29*	MIMAT0004780
**hsa-miR-18b-5p**	4.56E-06	1.79E-07	down	-79.16*	MIMAT0001412
**hsa-miR-362-3p**	4.31E-06	1.30E-07	down	-79.51*	MIMAT0004683
**hsa-miR-33a-5p**	7.76E-05	1.05E-05	down	-81.28*	MIMAT0000091
**hsa-miR-185-5p**	1.21E-05	8.20E-07	down	-89.77*	MIMAT0000455
**hsa-miR-151a-3p**	2.98E-05	2.92E-06	down	-97.29*	MIMAT0000757
**hsa-miR-362-5p**	3.25E-05	3.42E-06	down	-103.52*	MIMAT0000705
**hsa-miR-151b**	4.31E-06	8.82E-08	down	-105.68*	MIMAT0010214
**hsa-miR-194-5p**	6.64E-05	8.49E-06	down	-170.05*	MIMAT0000460
**hsa-miR-532-5p**	1.14E-05	5.81E-07	down	-190.43*	MIMAT0002888
**hsa-miR-660-5p**	4.31E-06	1.28E-07	down	-192.44*	MIMAT0003338
**hsa-miR-18a-5p**	4.31E-06	1.28E-07	down	-221.76*	MIMAT0000072
**hsa-miR-215-5p**	1.69E-05	1.21E-06	down	-1029.11*	MIMAT0000272

* miRNAs not detected in T48B or **T47B.

Supplementary Table S7: **Significantly enriched Gene Ontology biological process terms**

**Gene ontology biological process terms**	**Number of genes**	***p* value**	**adj. *p* value**
positive regulation of metabolic process	359	1.53E-68	1.53E-66
cell proliferation	290	2.05E-65	1.03E-63
positive regulation of cellular metabolic process	340	7.54E-65	2.51E-63

regulation of developmental process	283	2.10E-62	5.25E-61
regulation of cell proliferation	242	6.23E-62	1.25E-60
positive regulation of developmental process	174	8.61E-58	1.44E-56
regulation of cell differentiation	222	1.89E-57	2.70E-56
positive regulation of transcription from RNA polymerase II promoter	167	5.24E-54	6.55E-53
regulation of transcription from RNA polymerase II promoter	245	1.42E-53	1.40E-52
positive regulation of transcription, DNA-dependent	213	1.54E-53	1.40E-52
positive regulation of cellular process	213	1.54E-53	1.40E-52
regulation of multicellular organismal process	472	3.96E-53	3.30E-52
positive regulation of nucleobase-containing compound metabolic process	314	2.24E-52	1.72E-51
positive regulation of RNA metabolic process	232	4.67E-52	3.34E-51
regulation of programmed cell death	215	2.14E-50	1.43E-49
negative regulation of cellular process	233	2.42E-49	1.51E-48
positive regulation of cell differentiation	421	5.93E-49	3.49E-48
regulation of apoptotic process	133	2.93E-48	1.63E-47
negative regulation of biological process	229	6.15E-48	3.24E-47
organ development	448	3.76E-47	1.88E-46
positive regulation of biological process	358	6.73E-45	3.20E-44
protein phosphorylation	495	6.95E-44	3.16E-43
transcription from RNA polymerase II promoter	216	4.82E-43	2.10E-42
phosphorylation	251	5.45E-42	2.27E-41
programmed cell death	230	9.75E-42	3.90E-41
apoptotic process	267	4.83E-41	1.86E-40
enzyme linked receptor protein signaling pathway	264	1.35E-40	4.82E-40
regulation of molecular function	264	1.35E-40	4.82E-40
negative regulation of programmed cell death	184	1.40E-40	4.83E-40
tissue development	273	1.63E-40	5.43E-40
regulation of cellular protein metabolic process	136	1.74E-40	5.61E-40
negative regulation of apoptotic process	228	2.22E-40	6.94E-40
regulation of protein metabolic process	217	3.47E-40	1.05E-39
regulation of phosphorylation	133	2.52E-39	7.20E-39
intracellular protein kinase cascade	133	2.52E-39	7.20E-39
positive regulation of signal transduction	237	3.94E-39	1.09E-38
tube development	171	7.64E-39	2.06E-38
negative regulation of cell proliferation	177	1.31E-38	3.45E-38
regulation of protein modification process	164	1.46E-38	3.74E-38
gland development	112	3.32E-38	8.30E-38
negative regulation of metabolic process	121	3.65E-38	8.90E-38
regulation of protein phosphorylation	186	5.72E-38	1.36E-37
response to organic substance	84	2.28E-36	5.30E-36

tissue morphogenesis	231	2.79E-36	6.34E-36
anatomical structure formation involved in morphogenesis	159	3.16E-36	7.02E-36
organ morphogenesis	282	5.75E-36	1.25E-35
cell development	115	1.85E-35	3.94E-35
positive regulation of cell proliferation	250	2.37E-35	4.94E-35
transmembrane receptor protein tyrosine kinase signaling pathway	155	4.69E-35	9.57E-35
response to abiotic stimulus	230	5.88E-35	1.18E-34
morphogenesis of an epithelium	137	9.75E-35	1.91E-34
regulation of signal transduction	136	2.28E-34	4.38E-34
hematopoietic or lymphoid organ development	144	1.22E-33	2.28E-33
cell migration	98	1.23E-33	2.28E-33
system development	272	1.26E-33	2.29E-33
positive regulation of protein metabolic process	124	2.08E-33	3.71E-33
hemopoiesis	160	2.87E-33	5.04E-33
growth	437	3.49E-33	6.02E-33
negative regulation of cellular metabolic process	162	6.35E-33	1.08E-32
vasculature development	119	8.27E-33	1.38E-32
immune system development	139	8.42E-33	1.38E-32
regulation of cellular component organization	211	9.49E-33	1.53E-32
anatomical structure morphogenesis	120	1.20E-32	1.90E-32
regulation of catalytic activity	127	1.54E-32	2.41E-32
regulation of cell cycle	199	1.86E-32	2.86E-32
positive regulation of response to stimulus	296	3.02E-32	4.58E-32
response to external stimulus	215	4.53E-32	6.76E-32
regulation of cell migration	142	6.29E-32	9.25E-32
positive regulation of cell migration	200	8.61E-32	1.25E-31
intracellular signal transduction	196	1.47E-31	2.10E-31
regulation of kinase activity	96	7.31E-31	1.02E-30
response to endogenous stimulus	72	7.36E-31	1.02E-30
negative regulation of transcription, DNA-dependent	266	1.15E-30	1.57E-30
negative regulation of biosynthetic process	126	1.16E-30	1.57E-30
regulation of transferase activity	182	1.21E-30	1.61E-30
regulation of growth	148	4.71E-30	6.12E-30
positive regulation of cellular protein metabolic process	148	4.71E-30	6.12E-30
response to wounding	170	8.15E-30	1.04E-29
negative regulation of cellular biosynthetic process	127	8.29E-30	1.05E-29
regulation of response to stimulus	104	2.07E-29	2.59E-29
epithelial cell differentiation	145	2.28E-29	2.81E-29
negative regulation of RNA metabolic process	175	2.59E-29	3.16E-29
negative regulation of developmental process	167	3.58E-29	4.31E-29
negative regulation of nucleobase-containing compound metabolic process	325	5.20E-29	6.19E-29

regulation of gene expression	79	1.39E-28	1.64E-28
positive regulation of protein modification process	148	2.72E-28	3.16E-28
tube morphogenesis	115	3.46E-28	3.98E-28
regulation of protein kinase activity	157	4.32E-28	4.91E-28
regulation of cellular metabolic process	394	6.62E-28	7.44E-28
cell surface receptor signaling pathway	133	6.76E-28	7.51E-28
neurogenesis	79	7.44E-28	8.18E-28
angiogenesis	116	2.21E-27	2.40E-27
positive regulation of phosphate metabolic process	489	2.49E-27	2.68E-27
response to chemical stimulus	319	2.53E-27	2.69E-27
positive regulation of phosphorylation	177	3.44E-27	3.62E-27
negative regulation of cell differentiation	87	5.38E-27	5.60E-27

Supplementary Table S8: **Significantly enriched KEGG pathways**

**Gene ontology biological process terms**	**Number of genes**	***p* value**	**adj. *p* value**
Pathways in cancer	116	1.63E-45	1.63E-43
Prostate cancer	45	1.62E-24	8.10E-23
Chronic myeloid leukemia	41	2.94E-24	9.80E-23
Pancreatic cancer	37	4.99E-21	1.25E-19
Colorectal cancer	29	2.60E-18	5.20E-17
HTLV-I infection	60	1.14E-17	1.90E-16
Melanoma	33	3.95E-17	5.64E-16
Bladder cancer	21	3.05E-16	3.81E-15
Glioma	31	6.95E-16	7.72E-15
Focal adhesion	57	1.40E-15	1.40E-14
Neurotrophin signaling pathway	43	1.80E-15	1.64E-14
ErbB signaling pathway	35	6.11E-15	5.09E-14
Acute myeloid leukemia	28	7.09E-15	5.45E-14
TGF-beta signaling pathway	34	1.22E-14	8.71E-14
Non-small cell lung cancer	26	3.92E-14	2.61E-13
Osteoclast differentiation	40	8.37E-14	5.23E-13
MAPK signaling pathway	64	1.33E-13	7.82E-13
Cell cycle	40	3.93E-13	2.18E-12
Toxoplasmosis	34	4.22E-13	2.22E-12
Chagas disease (American trypanosomiasis)	33	5.97E-13	2.99E-12
Renal cell carcinoma	26	2.72E-12	1.30E-11
Measles	34	8.85E-12	4.02E-11
p53 signaling pathway	27	1.25E-11	5.43E-11
Hepatitis C	33	2.48E-11	1.03E-10
Adherens junction	27	2.81E-11	1.12E-10
Small cell lung cancer	29	3.07E-11	1.18E-10
Endometrial cancer	21	3.78E-11	1.40E-10

Epstein-Barr virus infection	29	1.08E-09	3.86E-09
Thyroid cancer	15	3.29E-09	1.13E-08
Toll-like receptor signaling pathway	29	5.70E-09	1.90E-08
Leishmaniasis	20	8.02E-09	2.59E-08
Jak-STAT signaling pathway	29	9.59E-09	3.00E-08
Influenza A	30	1.64E-08	4.97E-08
T cell receptor signaling pathway	28	3.19E-08	9.38E-08
Wnt signaling pathway	35	5.78E-08	1.65E-07
Cytokine-cytokine receptor interaction	50	1.18E-07	3.28E-07
Regulation of actin cytoskeleton	40	1.26E-07	3.41E-07
Insulin signaling pathway	32	5.80E-07	1.53E-06
B cell receptor signaling pathway	22	6.01E-07	1.54E-06
mTOR signaling pathway	16	1.25E-06	3.13E-06
Dorso-ventral axis formation	8	1.89E-06	4.61E-06
Tuberculosis	36	2.58E-06	6.14E-06
VEGF signaling pathway	21	3.19E-06	7.42E-06
Adipocytokine signaling pathway	18	9.73E-06	0.000022
Fc epsilon RI signaling pathway	20	9.9E-06	0.000022
GnRH signaling pathway	23	1.03E-05	2.24E-05
Pertussis	16	1.08E-05	2.3E-05
Endocytosis	24	1.14E-05	2.38E-05
Apoptosis	21	1.45E-05	2.96E-05
Herpes simplex infection	24	1.62E-05	3.24E-05
Type II diabetes mellitus	15	1.65E-05	3.24E-05
Chemokine signaling pathway	36	1.86E-05	3.58E-05
Progesterone-mediated oocyte maturation	20	0.000028	5.28E-05
Axon guidance	25	6.06E-05	0.000112
Epithelial cell signaling in Helicobacter pylori infection	12	7.81E-05	0.000142
Rheumatoid arthritis	8	0.00016	0.000286
Legionellosis	12	0.000182	0.000319
Salmonella infection	17	0.000237	0.000409
Melanogenesis	21	0.000309	0.000524
NOD-like receptor signaling pathway	13	0.000382	0.000637
Gap junction	19	0.000418	0.000685
Fc gamma R-mediated phagocytosis	20	0.00048	0.000774
Basal cell carcinoma	12	0.000929	0.001475
African trypanosomiasis	8	0.00138	0.002156
Long-term potentiation	15	0.0016	0.002462
Malaria	5	0.00197	0.002985
Cholinergic synapse	18	0.00251	0.003746
Aldosterone-regulated sodium reabsorption	9	0.00307	0.004515
Arrhythmogenic right ventricular cardiomyopathy (ARVC)	5	0.00312	0.004522
Shigellosis	11	0.00319	0.004557
Natural killer cell mediated cytotoxicity	23	0.00389	0.005479
Bacterial invasion of epithelial cells	12	0.00459	0.006288
Leukocyte transendothelial migration	19	0.00459	0.006288

Transcriptional misregulation in cancer	6	0.00598	0.008081
Amyotrophic lateral sclerosis (ALS)	9	0.00817	0.010893
Tight junction	19	0.012	0.015789
Hedgehog signaling pathway	11	0.0127	0.016494
Carbohydrate digestion and absorption	5	0.0212	0.027179
Notch signaling pathway	9	0.0271	0.034304
Viral myocarditis	6	0.0291	0.036375
Cytosolic DNA-sensing pathway	5	0.0328	0.040494
RIG-I-like receptor signaling pathway	9	0.0346	0.042195
Prion diseases	5	0.0399	0.048072
Oocyte meiosis	16	0.0406	0.048333
