# Supplementary figures and images for: Role of N-Cadherin in Epithelial-to-Mesenchymal Transition and Chemosensitivity of Colon Carcinoma Cells
Source: Cancers (Basel). 2022 Oct 20;14(20):5146. doi: 10.3390/cancers14205146 (PMC9601123; doi:10.3390/cancers14205146)

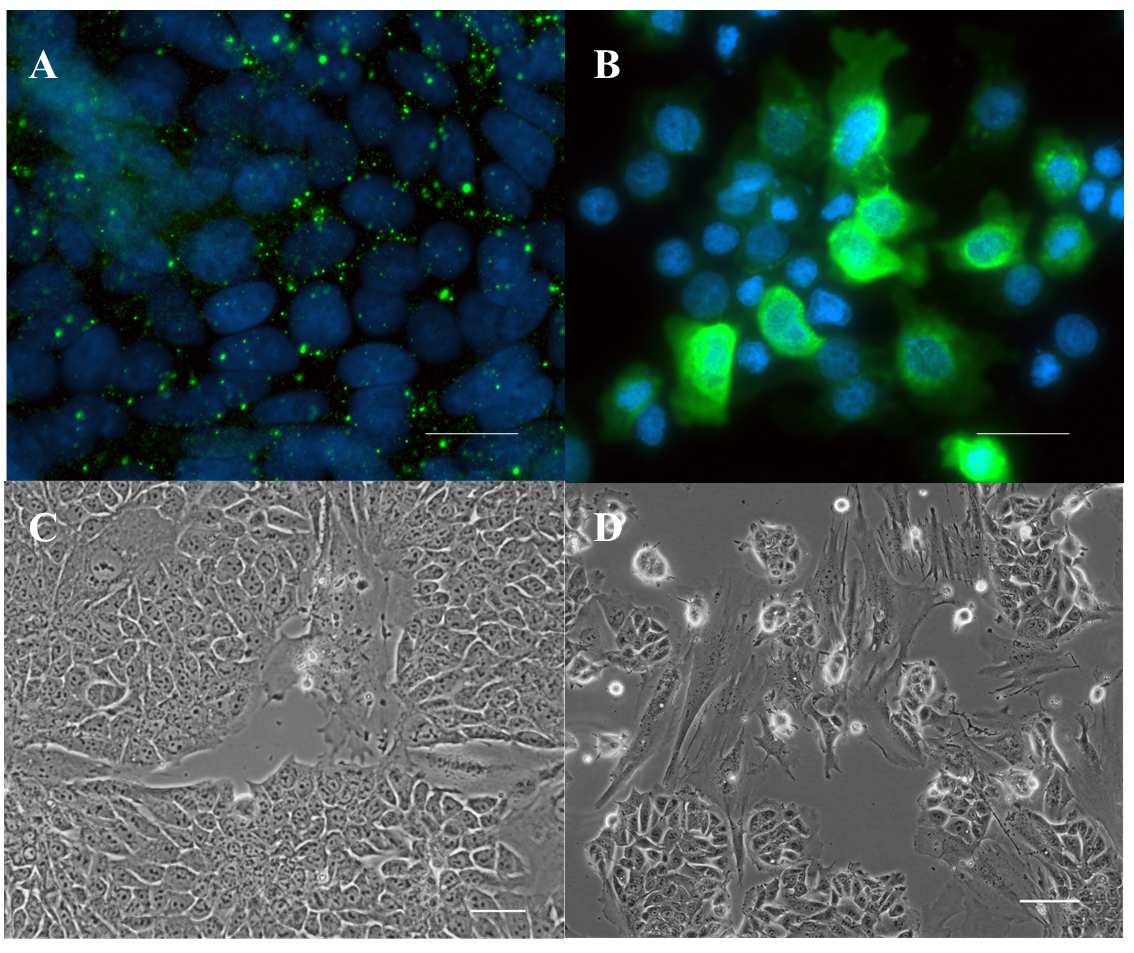

Supplement: Supplementary file 1 [file cancers-14-05146-s001.zip › Fig 1-S.tif]

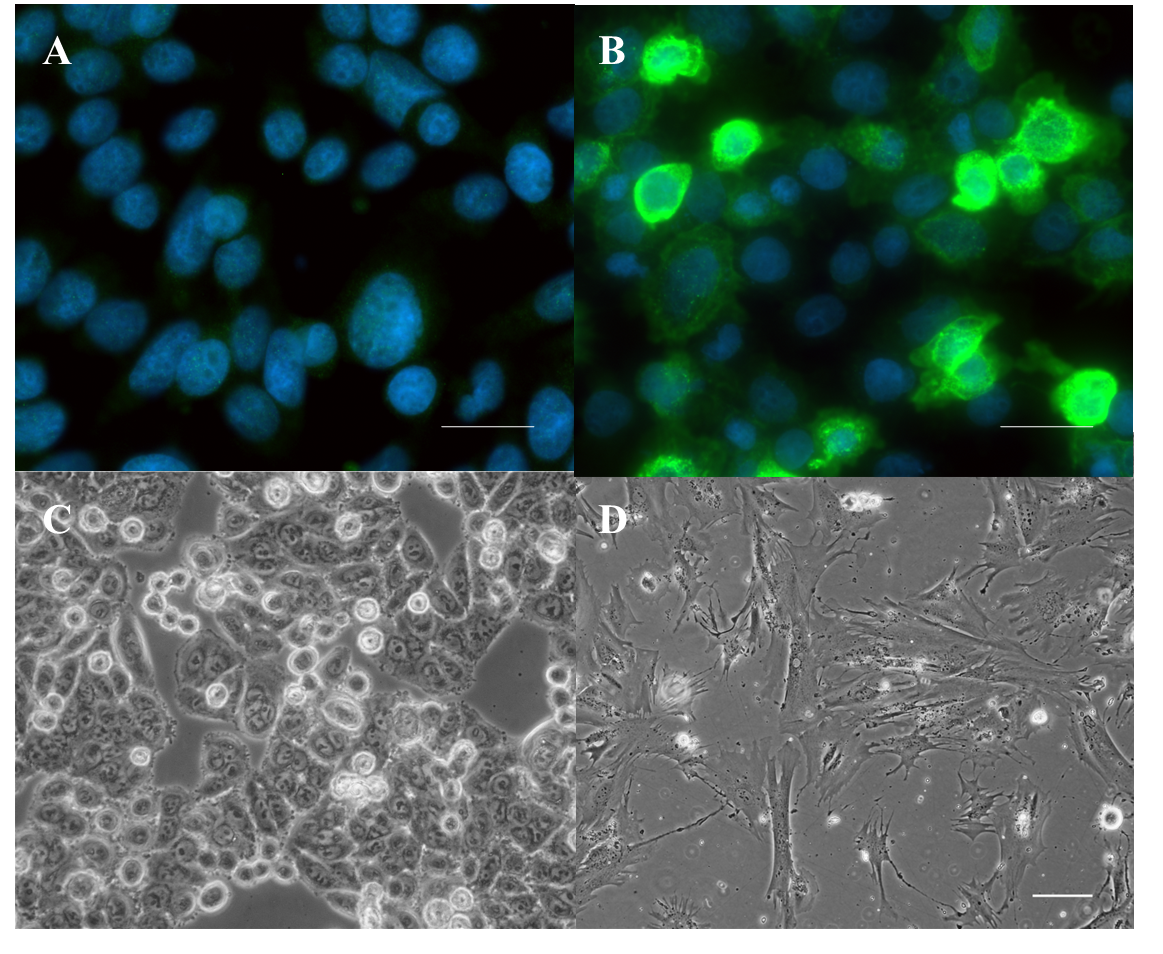

Supplement: Supplementary file 1 [file cancers-14-05146-s001.zip › Fig 2-S.tif]

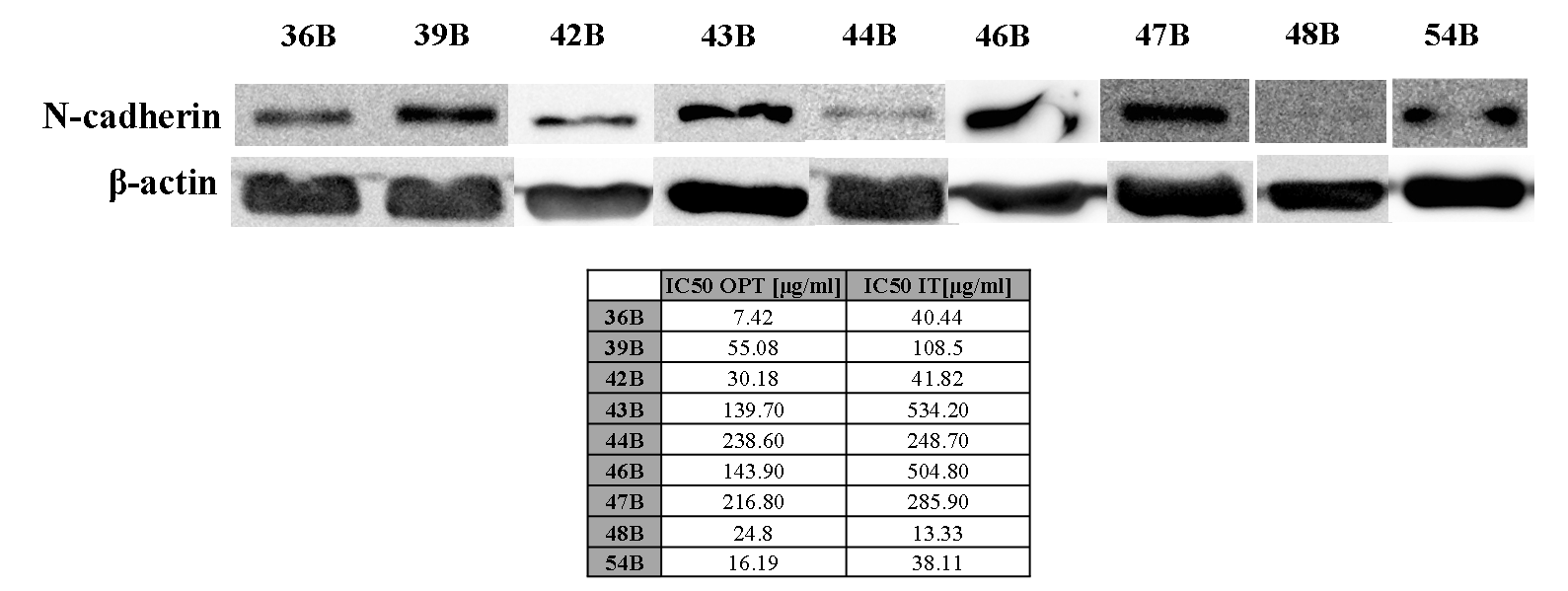

Supplement: Supplementary file 1 [file cancers-14-05146-s001.zip › Fig 3-S.tif]

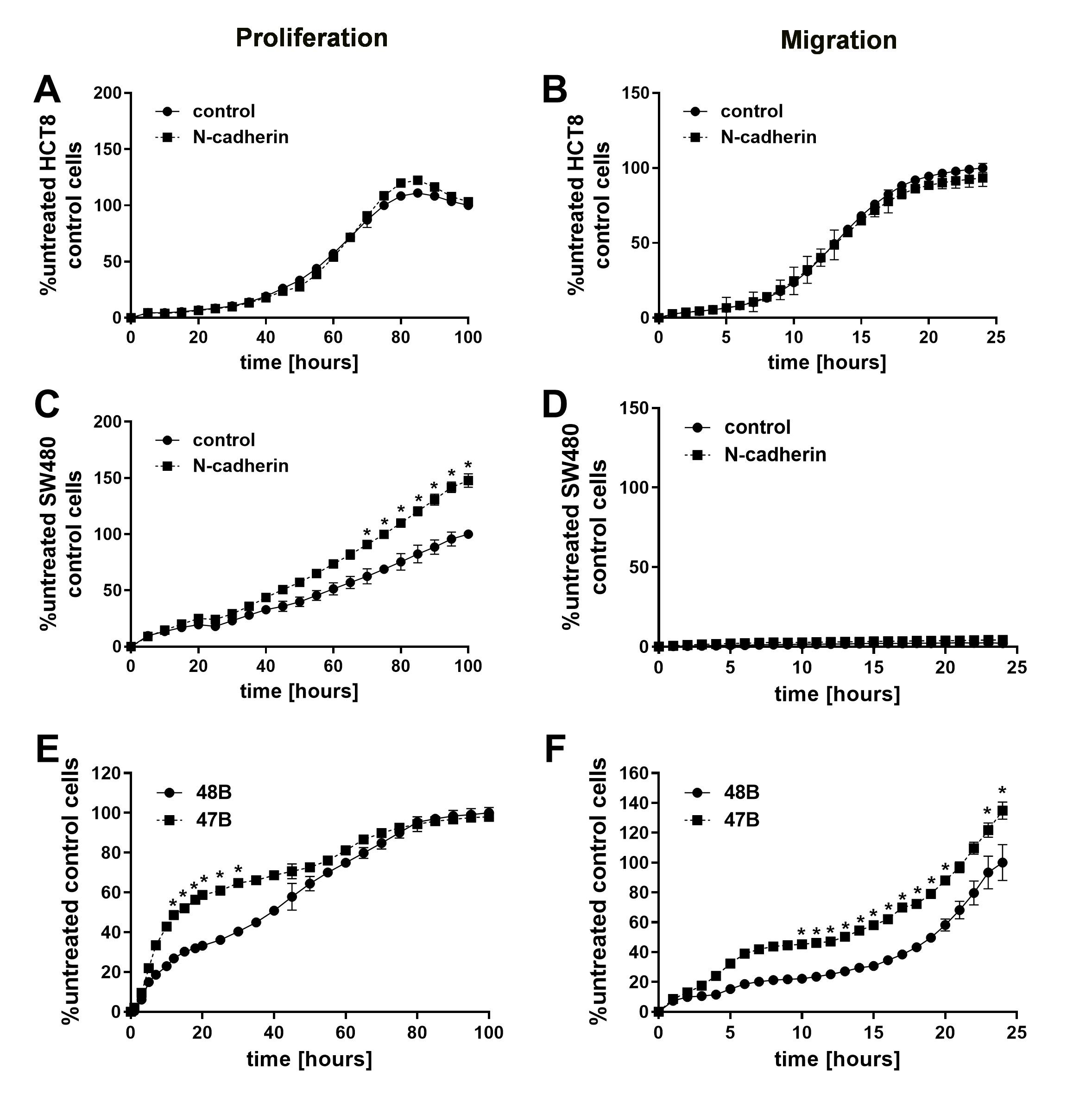

Supplement: Supplementary file 1 [file cancers-14-05146-s001.zip › Fig 4-S revised.tif]

Western blots

1st repetition

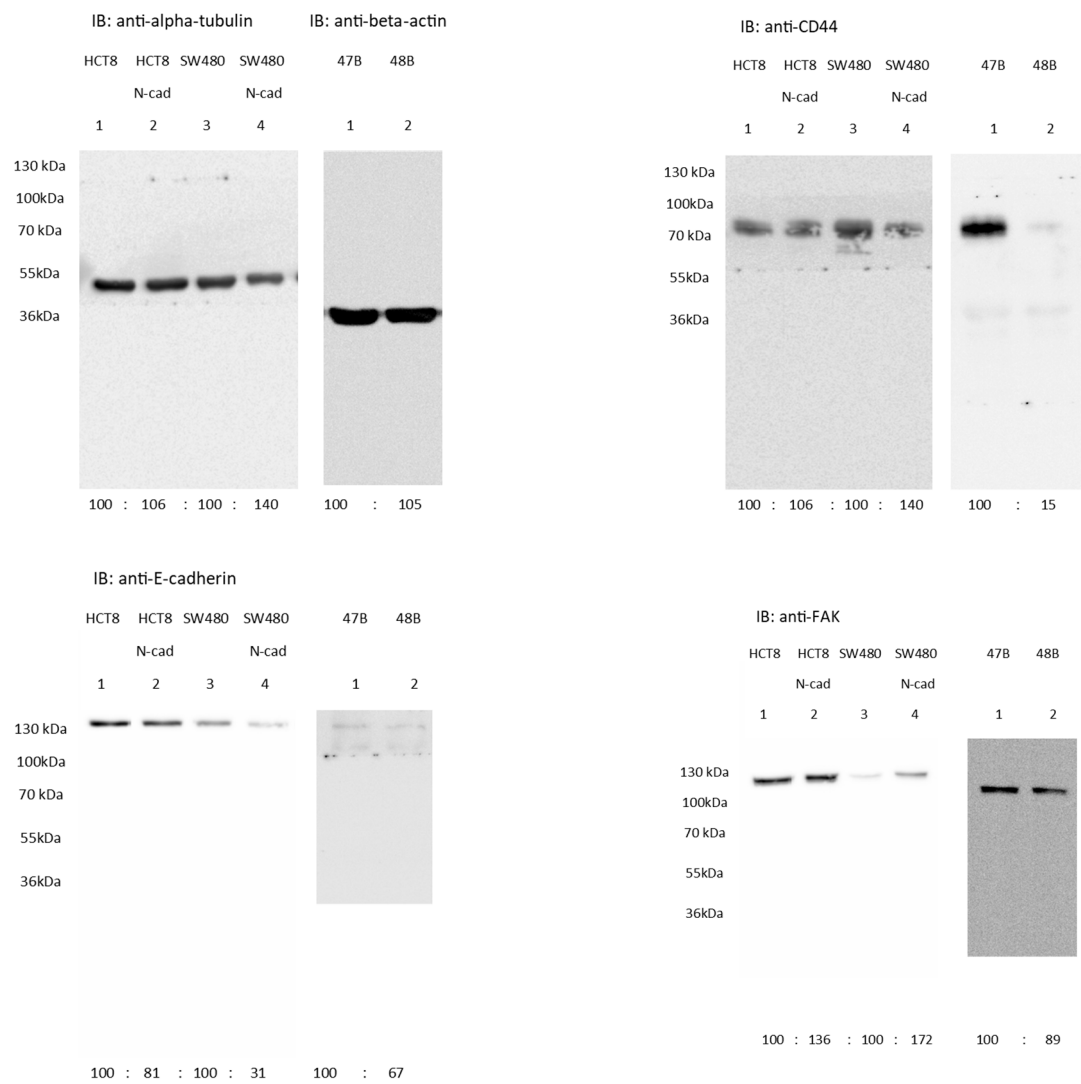

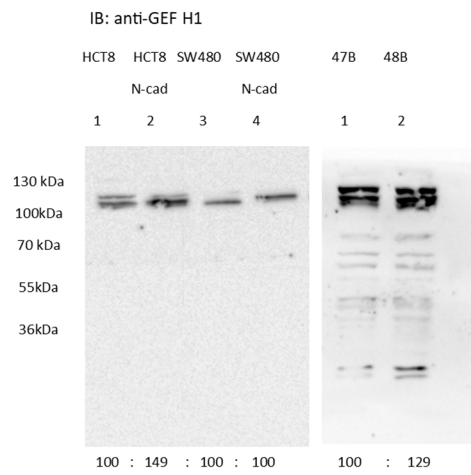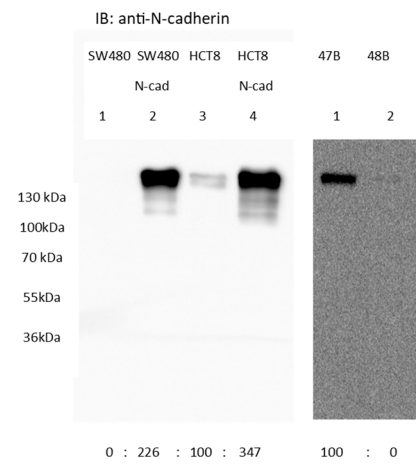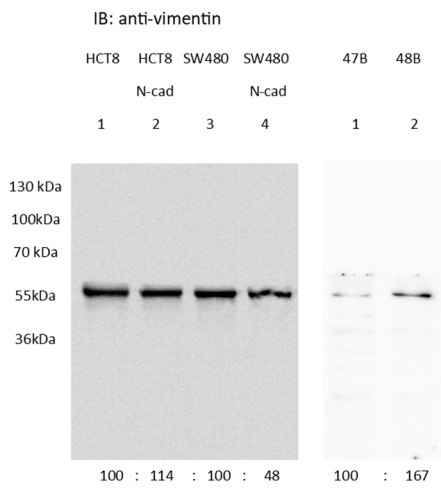

2nd repetition

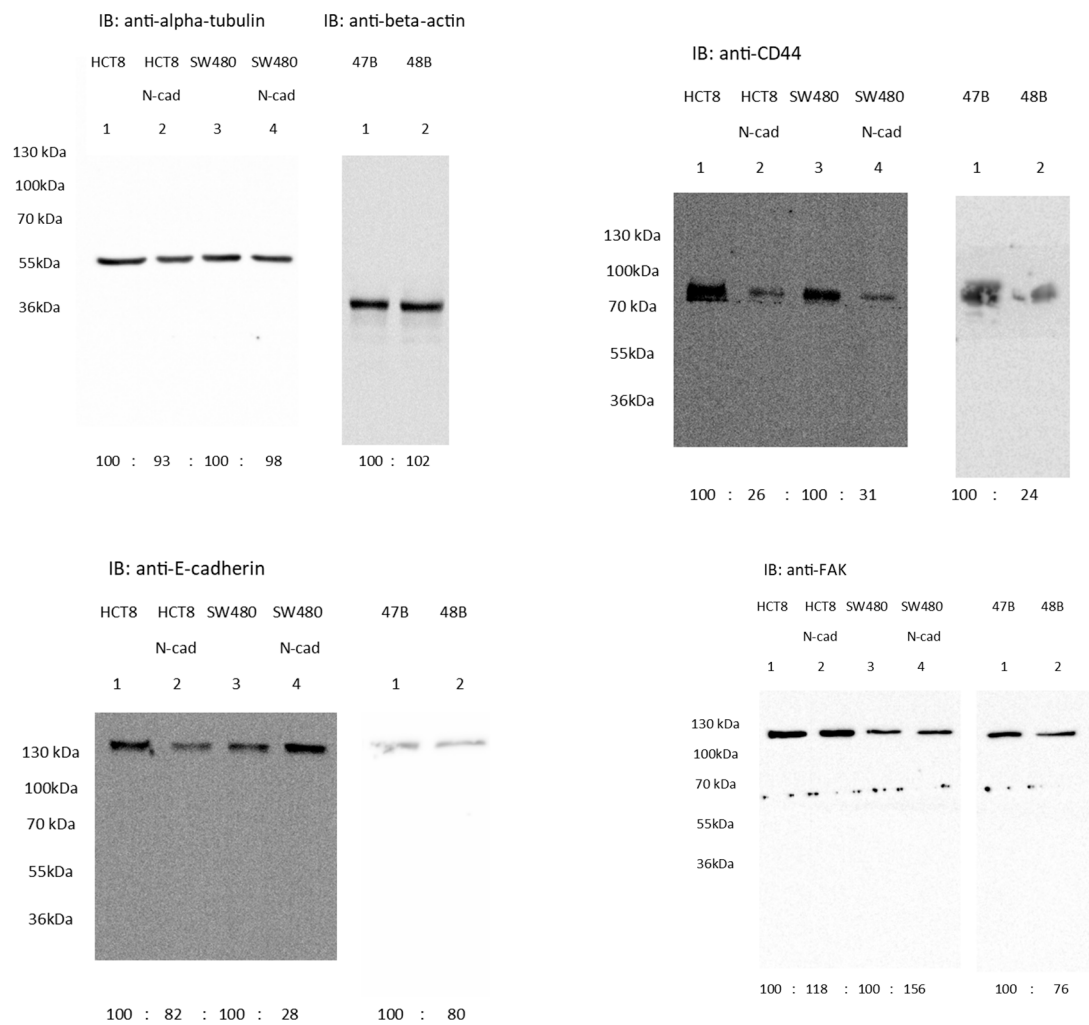

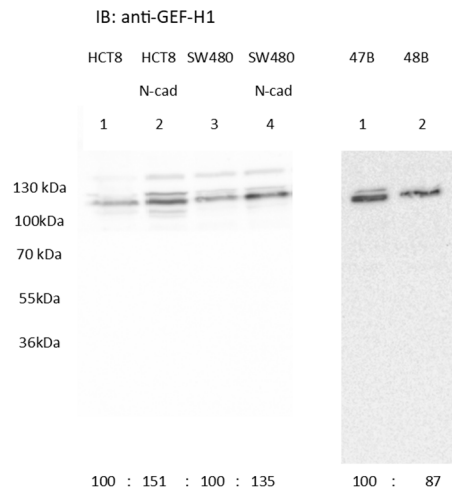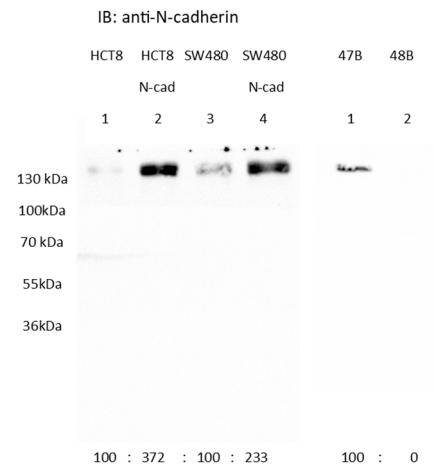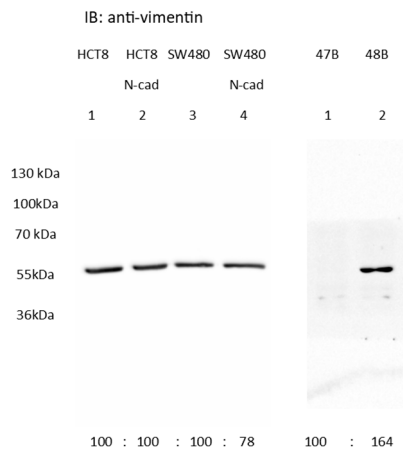

3rd repetition

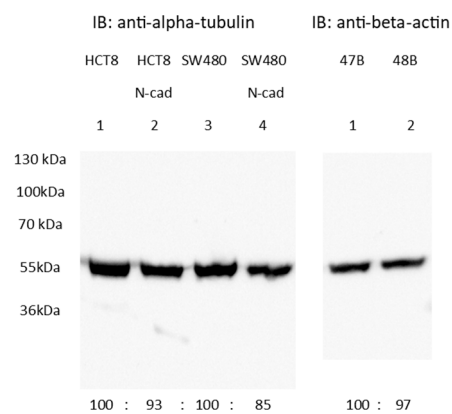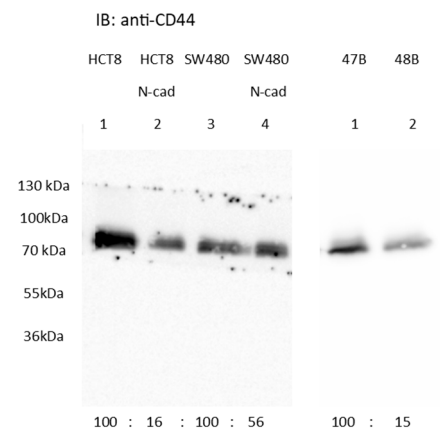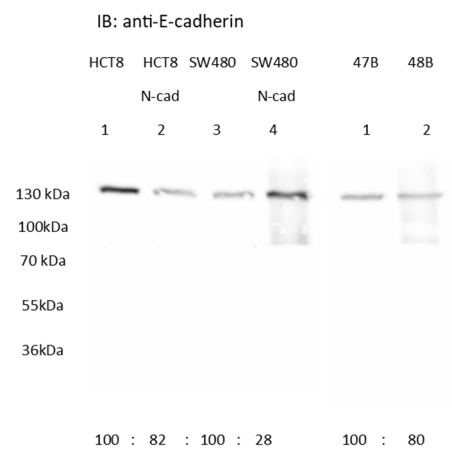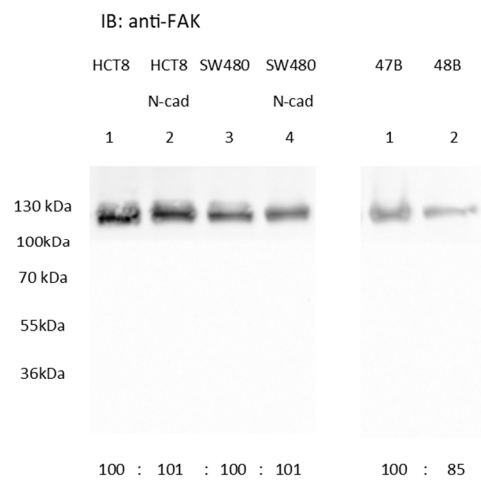

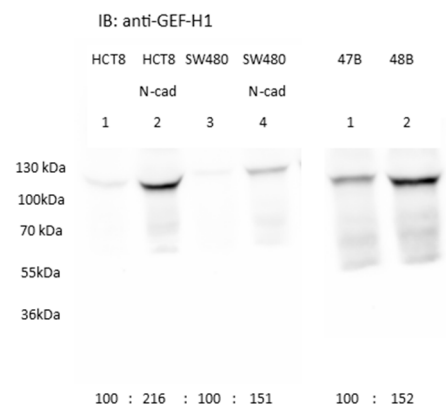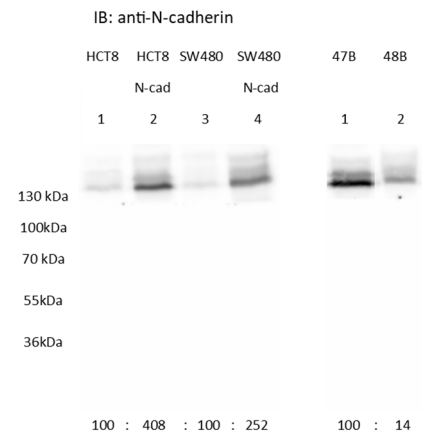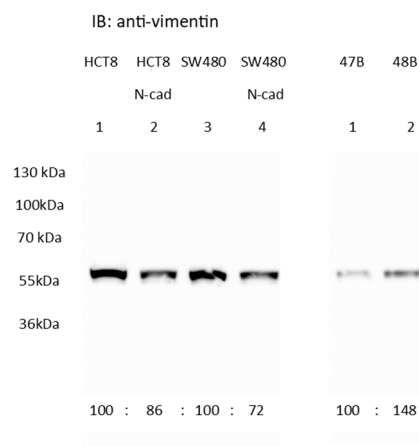

Supplement: Supplementary file 1 [file cancers-14-05146-s001.zip › File S1. WB - original blots with density.pdf]
